# Draft Genome Sequence of *Pseudomonas stutzeri* LH-42, Isolated from Petroleum-Contaminated Soil

**DOI:** 10.1128/genomeA.00589-17

**Published:** 2017-07-06

**Authors:** Mengjun Zhang, Liyuan Ma, Yu Yang, Tingting Hu, Xueduan Liu, Xian Zhang, Yaokun Hua, Yu Gao, Zhenyu Zhu

**Affiliations:** School of Minerals Processing and Bioengineering, Central South University, Hunan Province, China

## Abstract

We used Illumina HiSeq technology to sequence the whole genome of *Pseudomonas stutzeri* LH-42, a bacterium isolated from petroleum-contaminated soil in Liaoning Province, China. The bacterium contains a series of specific genes involved in the oxidation of organic sulfur compounds.

## GENOME ANNOUNCEMENT

The *Pseudomonas* genus belongs to the gamma subgroup of *Proteobacteria*, and *Pseudomonas stutzeri* is a characterized heterotroph known to degrade several hydrocarbons (1, 2). It has commonly been detected in petroleum-contaminated soil, and it degrades organic sulfur compounds, such as dibenzothiophene (DBT), in specific pathways. To investigate the mechanism of *P. stutzeri* that enables it to survive and proliferate in an oilfield environment and to further research sulfur metabolism, we determined the draft genome sequence of *P. stutzeri* LH-42. The genome contains genes involved in a broad utilization of carbon sources, nitrogen fixation, denitrification, sulfur metabolism, degradation of aromatic compounds, iron acquisition and metabolism, multiple pathways of protection against environmental stress, and other functions that presumably give LH-42 an advantage in root colonization.

The strain *P. stutzeri* LH-42 was isolated from petroleum-contaminated soil collected from Liaohe Oil Field, Liaoning, China. This bacterium was cultivated with basal salt medium (BSM), the components of which were as follows: 2.44 g/liter KH_2_PO_4_, 14.03 g/liter Na_2_HPO_4_·12H_2_O, 0.36 g/liter MgCl_2_·6H_2_O, 2.00 g/liter NH_4_Cl, 0.004 g/liter MnCl_2_·4H_2_O, 0.001 g/liter FeCl_3_·6H_2_O, 0.001 g/liter CaCl_2_, and 1.62 g/liter glycerol, and dibenzothiophene (DBT) was used as the sole electron donor (30°C at 170 rpm). The total genomic DNA of strain LH-42 was extracted using the TIANamp bacteria DNA kit (Tiangen, Beijing, China), and the whole genome was sequenced using an Illumina HiSeq 2000 (Illumina, San Diego, CA). The genome sequence assembly was performed using SOAP*denovo* (3), and coding sequences (CDSs) were predicted using Glimmer (4). Functional annotation was performed through homologous comparison of each putative gene against the public databases, including the nonredundant (NR) protein database, KEGG, and COG using BLAST. In addition, RNAmmer and tRNAscan-SE were used to identify rRNAs and tRNAs, respectively.

The draft genome of *P. stutzeri* LH-42 contains a total of 4,477,807 bp distributed in 298 contigs, with a G+C content of 64.06%. The draft genome has an *N*_50_ and *N*_90_ of 27,476 and 7,192 bp, respectively. The annotation results revealed one 5S-16S-23S operon, 93 tRNAs, and 4,413 CDSs, of which 66% (2,913 CDSs) were annotated as known proteins in the public database, 29% (1,280 CDSs) were conserved hypothetical proteins, and 5% (220 CDSs) were hypothetical proteins.

A number of genes potentially responsible for the oxidation of inorganic sulfur were identified: a dissimilatory sulfite reductase gene (*dsr*), an adenylyl-sulfate reductase (APSR) gene, and a phosphoadenylyl-sulfate reductase (PAPSR) gene, but the DBT monooxygenase gene (*dsz*) was not detected, which indicated that the *P. stutzeri* LH-42 metabolized the organic sulfur in the Kodama metabolic pathway. A complete whole-genome analysis is in progress, with an expectation to reveal additional information on the catabolic potential of *P. stutzeri* LH-42.

### Accession number(s).

This whole-genome shotgun project has been deposited at DDBJ/EMBL/GenBank under the accession no. NCKS00000000. The version described in this paper is the second version, NCKS02000000.

## References

[1] Barathi S, Vasudevan N 2001 Utilization of petroleum hydrocarbons by Pseudomonas fluorescens isolated from a petroleum-contaminated soil. Environ Int 26:413–416. doi:10.1016/S0160-4120(01)00021-6. 11392760

[2] Lalucat J, Bennasar A, Bosch R, García-Valdés E, Palleroni NJ 2006 Biology of Pseudomonas stutzeri. Microbiol Mol Biol Rev 70:510–547. doi:10.1128/MMBR.00047-05. 16760312 PMC1489536

[3] Li R, Zhu H, Ruan J, Qian W, Fang X, Shi Z, Li Y, Li S, Shan G, Kristiansen K, Li S, Yang H, Wang J, Wang J 2010 De novo assembly of human genomes with massively parallel short read sequencing. Genome Res 20:265–272. doi:10.1101/gr.097261.109. 20019144 PMC2813482

[4] Delcher AL, Bratke KA, Powers EC, Salzberg SL 2007 Identifying bacterial genes and endosymbiont DNA with Glimmer. Bioinformatics 23:673–679. doi:10.1093/bioinformatics/btm009. 17237039 PMC2387122

